# CRISPR Interference-Based Inhibition of MAB_0055c Expression Alters Drug Sensitivity in Mycobacterium abscessus

**DOI:** 10.1128/spectrum.00631-23

**Published:** 2023-05-09

**Authors:** Thanh Quang Nguyen, Bo Eun Heo, Yujin Park, Seunghyeon Jeon, Arunima Choudhary, Cheol Moon, Jichan Jang

**Affiliations:** a Division of Life Science, Department of Bio & Medical Big Data (BK21 Four Program), Research Institute of Life Science, Gyeongsang National University, Jinju, Republic of Korea; b Department of Clinical Laboratory Science, Semyung University, Jecheon, Republic of Korea; University of Guelph College of Biological Science

**Keywords:** CRISPRi-dCAS9, LysR-type transcriptional regulators, Mycobacterium abscessus, rifabutin

## Abstract

There is an unmet medical need for effective treatments against Mycobacterium abscessus infections. Although advanced molecular genetic tools to validate drug targets and resistance of M. abscessus exist, the practical design and construction of plasmids are relatively laborious and time-consuming. Thus, for this purpose, we used CRISPR interference (CRISPRi) combined with catalytically deactivated Cas9 to inhibit the gene expression of a predicted LysR-type transcriptional regulator gene, *MAB_0055c*, in M. abscessus and evaluated its contribution to the development of drug resistance. Our results showed that silencing the MAB_0055c gene lead to increased rifamycin susceptibility depending on the hydroquinone moiety. These results demonstrate that CRISPRi is an excellent approach for studying drug resistance in M. abscessus.

**IMPORTANCE** In this study, we utilized CRISPR interference (CRISPRi) to specifically target the *MAB_0055c* gene in M. abscessus, a bacterium that causes difficult-to-treat infections. The study found that silencing the gene lead to increased rifabutin and rifalazil susceptibility. This study is the first to establish a link between the predicted LysR-type transcriptional regulator gene and antibiotic resistance in mycobacteria. These findings underscore the potential of using CRISPRi as a tool for elucidating resistance mechanisms, essential drug targets, and drug mechanisms of action, which could pave the way for more effective treatments for M. abscessus infections. The results of this study could have important implications for the development of new therapeutic options for this challenging-to-treat bacterial infection.

## INTRODUCTION

The Mycobacterium abscessus complex is regarded as the most chemotherapy-resistant species among nontuberculous mycobacteria (NTM) as it shows high drug resistance against current antibiotics, including antituberculosis agents, such as isoniazid and rifampicin (RIF) (1). Additionally, M. abscessus infections can be misdiagnosed as those of Mycobacterium tuberculosis, and the treatment of M. abscessus infections is challenging, prolonged, and expensive and often results in unsatisfactory outcomes (2, 3). Therefore, there is an urgent, unmet need for safe and effective agents against M. abscessus pulmonary disease (4).

RIF is one of the most well-known analogs of rifamycin. It exhibits bactericidal activity against both Gram-positive and Gram-negative bacteria by inhibiting RNA polymerase (5). Furthermore, RIF is used for the treatment of tuberculosis (TB), as a first-line anti-TB drug, as well as infections of NTMs (e.g., Mycobacterium kansasii and Mycobacterium avium complex) (6, 7). However, RIF is not utilized in the treatment of M. abscessus infections because of its poor *in vitro* activity (8–10). In stark contrast to RIF, rifabutin (RFB), another rifamycin analog, shows excellent anti-M. abscessus activity both *in vivo* and *in vitro*, owing to its favorable oral bioavailability and excellent pharmacokinetic properties, such as a long half-life and high cellular penetration. Furthermore, it reaches suitable concentrations in human lung tissue (8, 11, 12).

LysR-type transcriptional regulators (LTTRs) are considered to be the most numerous family of transcriptional regulators, with functional orthologs found in a wide range of bacterial genera, archaeal, and eukaryotic organisms (13). They control a variety of functions, including the regulation of metabolism and stress responses (14). Furthermore, LTTRs have been linked to many bacterial antibiotic resistance mechanisms in different pathogens, including Staphylococcus aureus and Klebsiella pneumoniae (15, 16). Interestingly, M. tuberculosis also contains the LTTR OxyS, which plays a role in the oxidative stress response. However, distinct from other pathogenic bacteria, the overproduction or depletion of OxyS in M. tuberculosis does not affect its susceptibility to developing resistance to drugs, such as isoniazid (17). In addition, Schneefeld et al. (18) demonstrated that another LTTR of M. tuberculosis, LysG (Rv1985c), activates the lysine exporter gene *lys*E(Rv1986) in the presence of lysine and histidine. Moreover, LysG controls genes that are involved in cell wall metabolism.

In recent years, new tools have emerged that enable researchers to explore the interaction between proteins with crucial functions and antibiotics in M. abscessus (19–21). One such tool is CRISPR interference (CRISPRi), which employs a deactivated Cas9 nuclease and a single-guide RNA to obstruct transcription at the targeted genomic locus. This approach has demonstrated effectiveness in knocking down both essential and nonessential genes. In this particular study, the CRISPRi assay was employed to verify the importance of MAB_0055c in M. abscessus growth, as well as to evaluate its contribution to susceptibility to antibiotics that are effective against M. abscessus.

## RESULTS AND DISCUSSION

Recently, we used the Mycomar T7 phagemid delivery system to generate transposon libraries in M. abscessus subsp. *abscessus* CIP 104536^T^ (unpublished data). Through this approach, we identified 19 genes that play an important role in the growth of M. abscessus in mouse bone-marrow-derived macrophages (mBMDMs). In this study, 1 of the 19 genes, namely, *MAB_0055c*, was evaluated to determine whether this LTTR contributes to M. abscessus drug resistance. For this evaluation, the CRISPR interference (CRISPRi) assay was utilized, enabling us to experimentally confirm the importance of MAB_0055c and to evaluate its contribution to antibiotics susceptibility. The plasmid pLJR962, containing an anhydrotetracycline (ATc)-inducible nuclease-deactivated *dCas9Sth1* (from Streptococcus thermophilus) and an ATc-inducible single guide RNA (sgRNA) along with a selection marker, was used as the vector for CRISPRi in M. abscessus as described previously (19). ATc-inducible (P_Tet_) dCas9 is directed to specific DNA targets by ATc-inducible or constitutively (P_con_) expressed sgRNA, preventing transcription initiation or elongation (21). ATc is a derivative of tetracycline that shows no antibiotic activity, and consequently, it is ideal for use in tetracycline-controlled gene expression (22).

**FIG 1:**
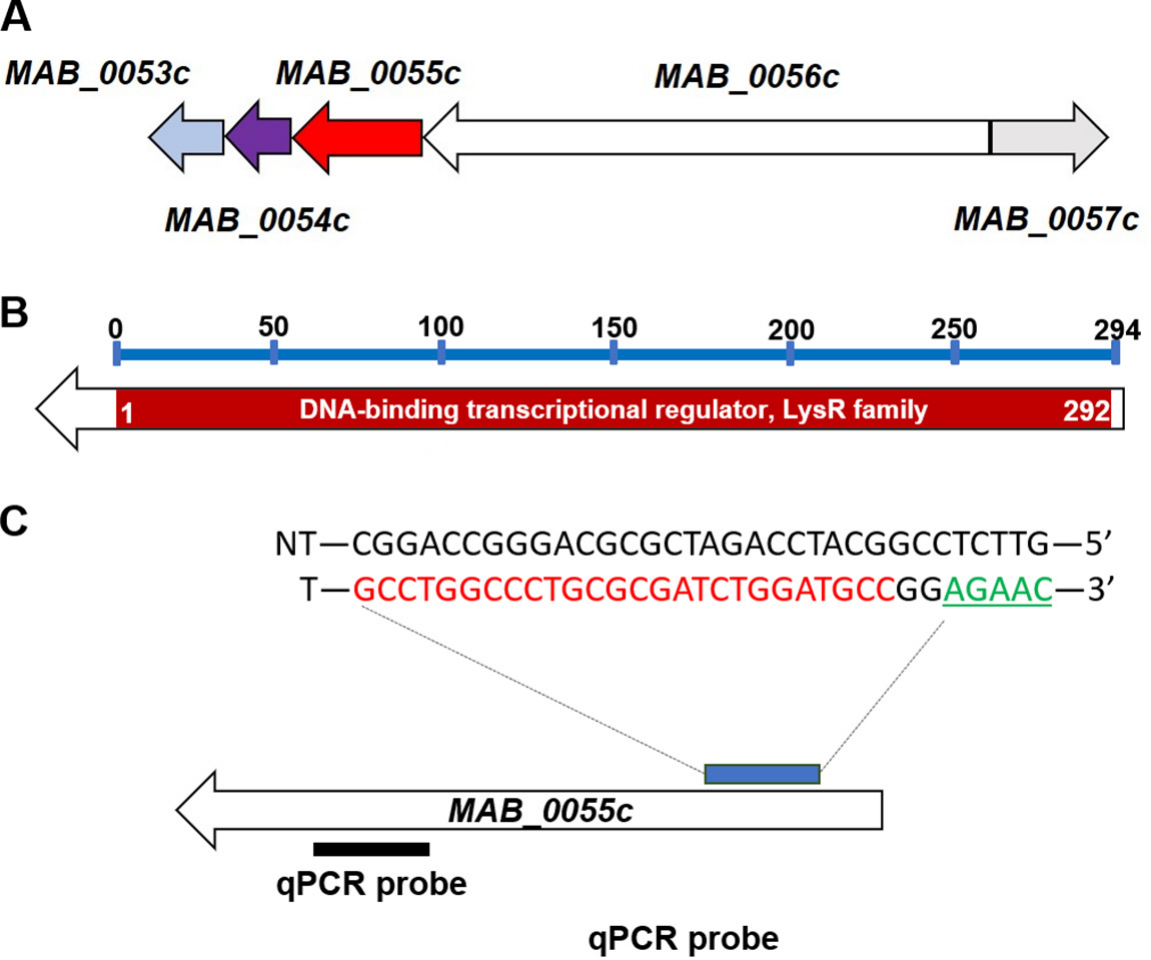
Genetic loci, domain map, and CRISPRi targeting of *MAB_0055c.* (A) Genetic organization at the *MAB_0055c* locus of M. abscessus. (B) Domain architecture of MAB_0055c. (C) PAM sequence (green text) and targeting sgRNA (blue text) used to conditionally inhibit *MAB_0055c* gene expression. The black bar represents the location of the real-time qPCR probe.

*MAB_0055c* is an 885-bp gene that encodes a 294-amino acid protein. It is hypothesized to be a member of the LysR-type transcriptional regulator (LTTR) family and is located adjacent to the probable cyanate hydratase gene (cyanase; MAB_0054c) (Fig. 1A). The first 292 amino acids of MAB_0055c correspond to the LysR substrate-binding domain (Fig. 1B). A protospacer adjacent motif (PAM) sequence was identified in *MAB_0055c*, and a 26-bp sequence complementary to the nontemplate target sequence was cloned into the pLJR962 upstream of the dCas9 as described previously (Fig. 1C) (21). To achieve the MAB_0055c-repressed M. abscessus strain, the pLJR962-*MAB_0055c* sgRNA constructs were electroporated into M. abscessus. Using a selected strain, we first quantified the transcriptional level of *MAB_0055c*-sgRNA under the presence or absence of ATc. After being cultured overnight, the *MAB_0055c*-sgRNA strain and an empty vector control (“empty”) were inoculated into 7H9 broth media, either with or without ATc, and real-time quantitative PCR (qPCR) was carried out using a probe (Fig. 1C). As shown in Fig. S1A in the supplemental material, no transcriptional changes were observed in either strain in the absence of ATc. However, the transcription level of *MAB_0055c* in the *MAB_0055c*-sgRNA strain was reduced by 38.01 ± 8.9% (mean ± standard deviation [SD]) in the presence of 150 ng/mL ATc compared with that of the empty vector control.

In general, when the target gene is in an operon, dCas9 will block the expression of all downstream genes, a phenomenon called a polar effect (23). In addition, its effect on upstream genes, also referred to as the reverse-polar effect, can be influenced by target gene interference depending on the organism (24). For this reason, we also tested the polar and reverse-polar effect of dCas9 when *MAB_0055c* transcription was inhibited by ATc. First, the transcriptional responses of an upstream gene, *MAB_0054c*, and a downstream gene, *MAB_0056c* (Fig. 1A), of the *MAB_0055c*-sgRNA strain were evaluated using real-time qPCR under the presence of ATc (150 ng/mL). As shown in Fig. S1B and C, expressions of *MAB_0054c* and *MAB_0056c*, respectively, were not influenced by ATc. Both genes showed the same RNA expression level as those of the empty vector control. Thus, the CRISPRi-dCas9 transcriptional inhibition impacted only the *MAB_0055c* gene and did not affect upstream or downstream genes.

**FIG 2:**
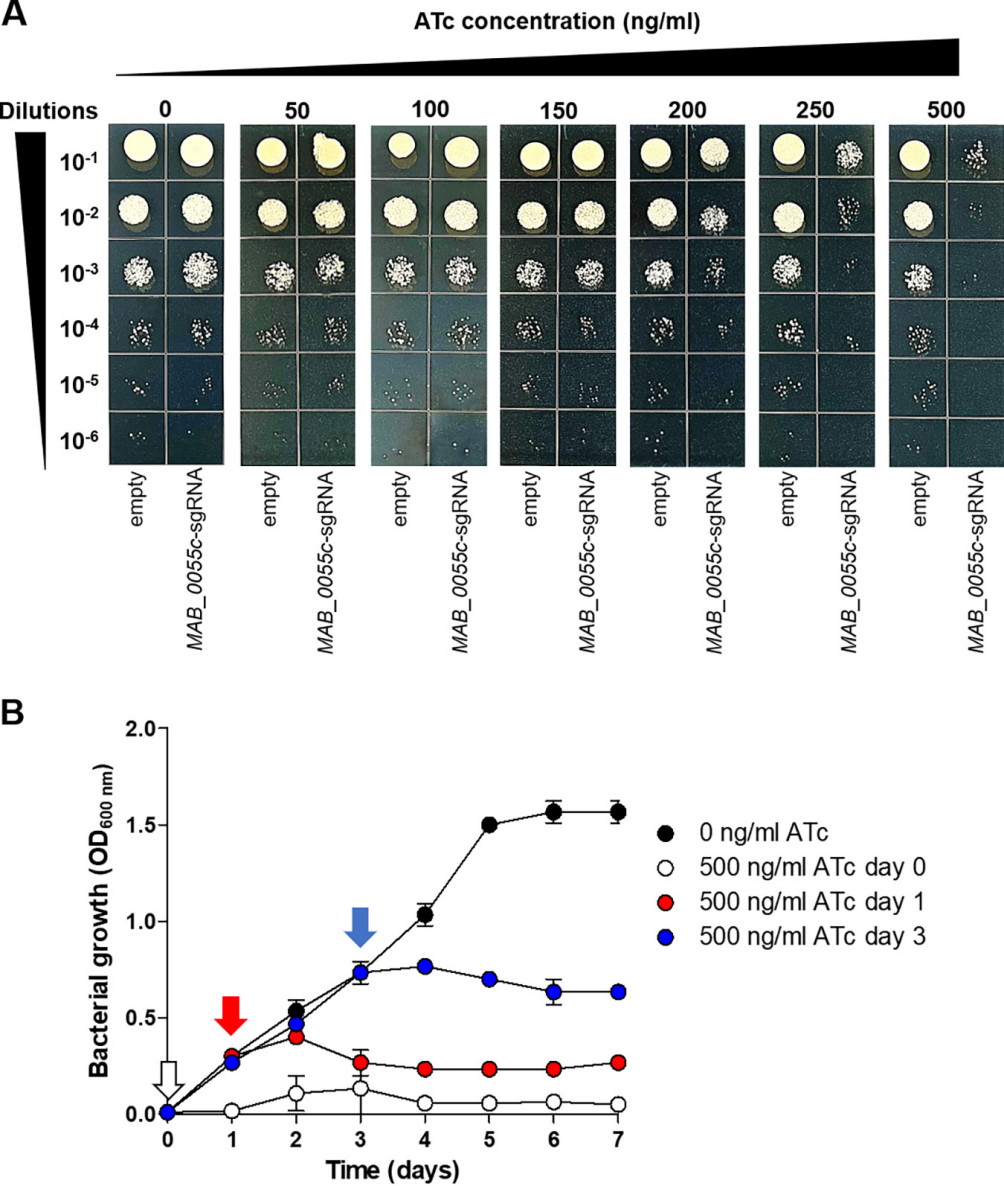
Growth of *MAB_0055c*-sgRNA strain under various ATc concentrations. (A) Diluted *MAB_0055c*-sgRNA strain culture and an empty vector control (“empty”) were serially diluted and plated onto 7H10 agar plates with ATc concentrations ranging from 0 to 500 ng/mL. (B) To the *MAB_0055c*-sgRNA strain cultures, 500 ng/mL ATc was added at day 0 (white arrow), 1 (red arrow), or 3 (blue arrow) from a starting OD_600_ of 0.005, with determinations of OD_600_.

Next, the importance of *MAB_0055c* for M. abscessus growth under different concentrations of ATc was evaluated. Briefly, the *MAB_0055c*-sgRNA strain and empty vector control cultures were prepared by dilution to an optical density at 600 nm (OD_600_) of approximately 0.2. The diluted culture was plated (5 μL) onto 7H10 agar plates supplemented with ATc at concentrations ranging from 0 to 500 ng/mL. As shown in Fig. 2A, the growth of the *MAB_0055c*-sgRNA strain is inhibited at ATc concentrations of ≥150 ng/mL. Above 200 ng/mL of ATc, the CFU reduction was dramatically increased compared with that of the empty vector control, confirming that MAB_0055c is required for *in vitro* growth. To test whether CRISPRi had an inhibitory effect against actively growing bacteria, the *MAB_0055c*-sgRNA strain was inoculated into 7H9 broth media and 500 ng/mL ATc was added to cultures at 0, 1, and 3 days postinoculation. At all 3 time points, we observed a cessation of bacterial growth at 2 days following the addition of ATc (Fig. 2B). For example, a 500-ng/mL ATc treatment at 3 days postinoculation resulted in an immediate growth stop and started to reduce the growth from day 5. Overall, these results demonstrate that mycobacterial CRISPRi produces a robust and proportional impairment of *MAB_0055c* gene expression, inhibiting mycobacterial growth.

**FIG 3:**
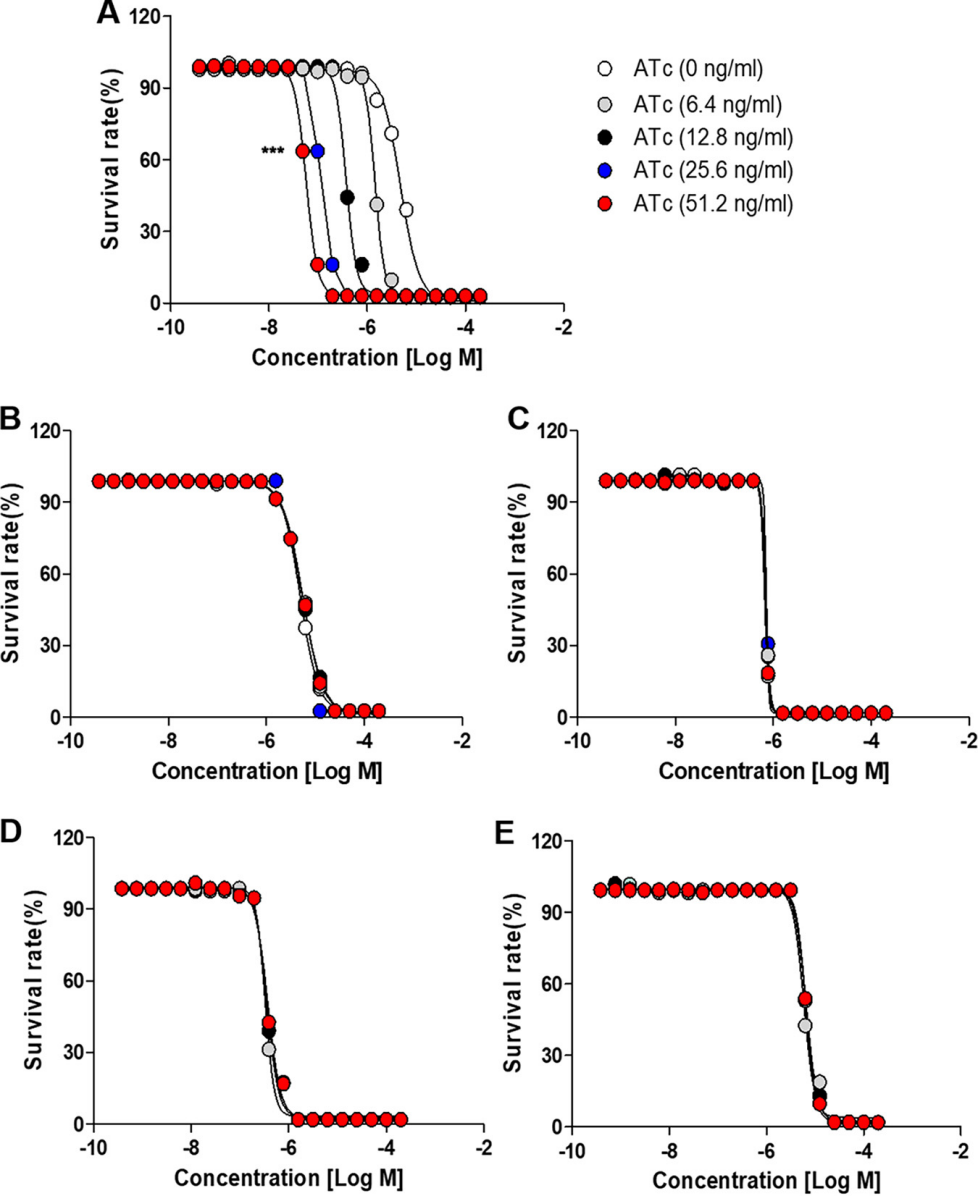
Interactions between ATc and various anti-M. abscessus drugs and their effects on the *MAB_0055c*-sgRNA strain. Several different anti-M. abscessus drugs were administered to *MAB_0055c*-sgRNA strains under various concentrations of ATc and following changes in drug antibiotic activity were assessed by dose-response curves for RFB (A), CLA (B), TGC (C), BDQ (D), and MFX (E). The survival of bacteria at each concentration was determined by calculating the ratio of relative light units (RLU) in the presence or absence of the inhibitor. The presented data are the means of three independent experiments and are displayed with standard deviation (SD) error bars. To determine statistical significance against the 0 ng/mL ATc control, a two-way analysis of variance (ANOVA) was conducted. The results showed a highly significant difference; ***, *P < *0.001.

**FIG 4:**
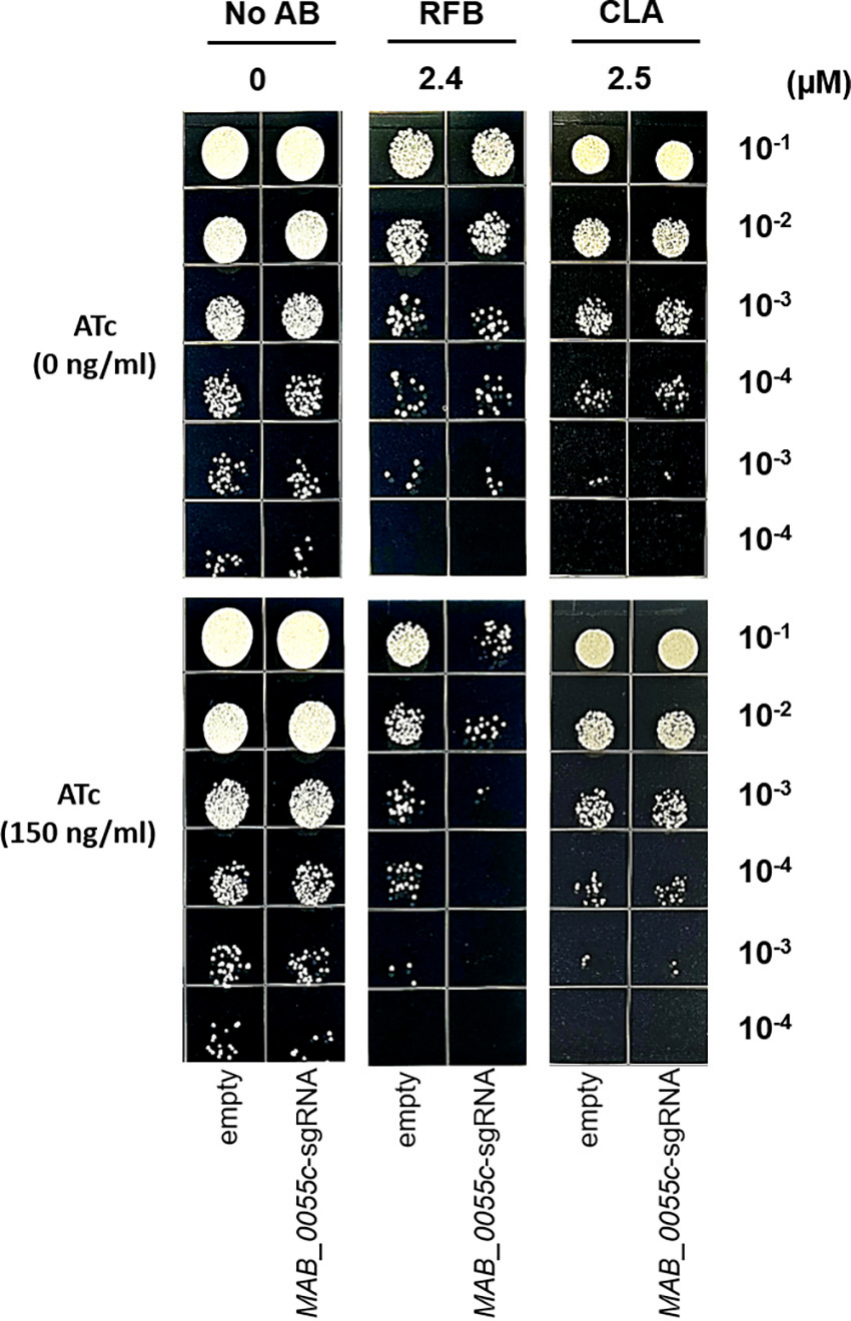
Interactions between ATc and either RFB or CLA and their effects on growth of the *MAB_0055c*-sgRNA strain. The *MAB_0055c*-sgRNA strains were treated with RFB and CLA in the absence and presence of ATc (150 ng/mL). Changes in the antibiotic activity of the drugs were assessed by measuring bacterial growth on agar plates. To do this measurement, aliquots of each culture were subjected to 10-fold serial dilution and spotted onto 7H10 agar plates containing either (+) or (−) ATc.

Since LTTRs are thought to be involved in the control of metabolism, stress response, cell division, virulence, secretion, and antibiotic resistance (25), we tested whether the repression of the LTTR MAB_0055c by CRISPRi-dCas9 contributes to the susceptibility of M. abscessus to antibiotics, such as RFB, clarithromycin (CLA), tigecycline (TGC), bedaquiline (BDQ), and moxifloxacin (MFX). Thus, the MIC at which 50% of the isolates tested are inhibited (MIC_50_) of those drugs was determined using a resazurin microtiter assay in a cation-adjusted Mueller-Hinton (CAMH) medium. In the absence of ATc, the MIC_50_s of RFB, CLA, TGC, BDQ, and MFX against the *MAB_0055c*-sgRNA strain were 4.8, 5.0, 0.7, 0.4, and 6.5 μM, respectively. To test whether inhibiting *MAB_0055c* gene expression affects M. abscessus susceptibility to antibiotics, the concentration of ATc was increased, ranging from 6.4 to 51.2 ng/mL, in a dose-dependent manner. ATc concentration does not reduce the growth of the *MAB_0055c*-sgRNA strain (data not shown). As shown in Fig. 3, RFB was the only antibiotic to show MIC_50_ changes in accordance with ATc concentrations. Specifically, increasing the dose of ATc (6.4 to 51.2 ng/mL) decreased the MIC_50_ of RFB against M. abscessus. For example, supplementation of the highest concentration of ATc at 51.2 ng/mL decreased the MIC_50_ of RFB by approximately 83.3-fold, while that of the other antibiotics tested did not change (Fig. 3B to E). This phenomenon was further evaluated using a 7H10 agar plate with and without ATc supplementation. RFB and CLA were tested at 0.5× MIC_50_. In Fig. 4, the 0.5× MIC_50_ of RFB (2.4 μM) significantly reduced bacterial (*MAB_0055c*-sgRNA strain) CFU in the presence of ATc at 150 ng/mL, compared with the empty vector control. Conversely, 0.5× MIC_50_ of CLA (2.5 μM) failed to reduce the CFU of the *MAB_0055c*-sgRNA strain. Other antibiotics, such as amikacin (AMK), TGC, and cefoxitin (CFX), showed no ATc-dependent effectiveness against the *MAB_0055c*-sgRNA strain as well (see Fig. S2 in the supplemental material). Thus, RFB showed MAB_0055c-specific dependence for its activity. In other words, MAB_0055c is specifically involved in RFB resistance of M. abscessus.

These *in vitro* RFB-specific MIC_50_ changes based on ATc concentrations were further analyzed using *MAB_0055c*-sgRNA strain-infected macrophages. The changes in RFB intracellular activity against the *MAB_0055c*-sgRNA strain, depending on ATc concentration, were evaluated in bone-marrow-derived macrophages (BMDMs). First, the intracellular influence of ATc on M. abscessus and the *MAB_0055c*-sgRNA strain in the absence of antibiotics was assessed. mWasabi protein-expressing M. abscessus- and *MAB_0055c*-sgRNA strain-infected macrophages were treated with various concentrations of ATc. As shown Fig. S3 in the supplemental material, ATc did not have an effect on either macrophage or M. abscessus survival (Fig. S3A). However, ATc treatment of the *MAB_0055c*-sgRNA strain-infected macrophages resulted in a decrease in intracellular bacterial number in a dose-dependent manner, while no change was observed in the number of macrophages (Fig. S3B).

**FIG 5:**
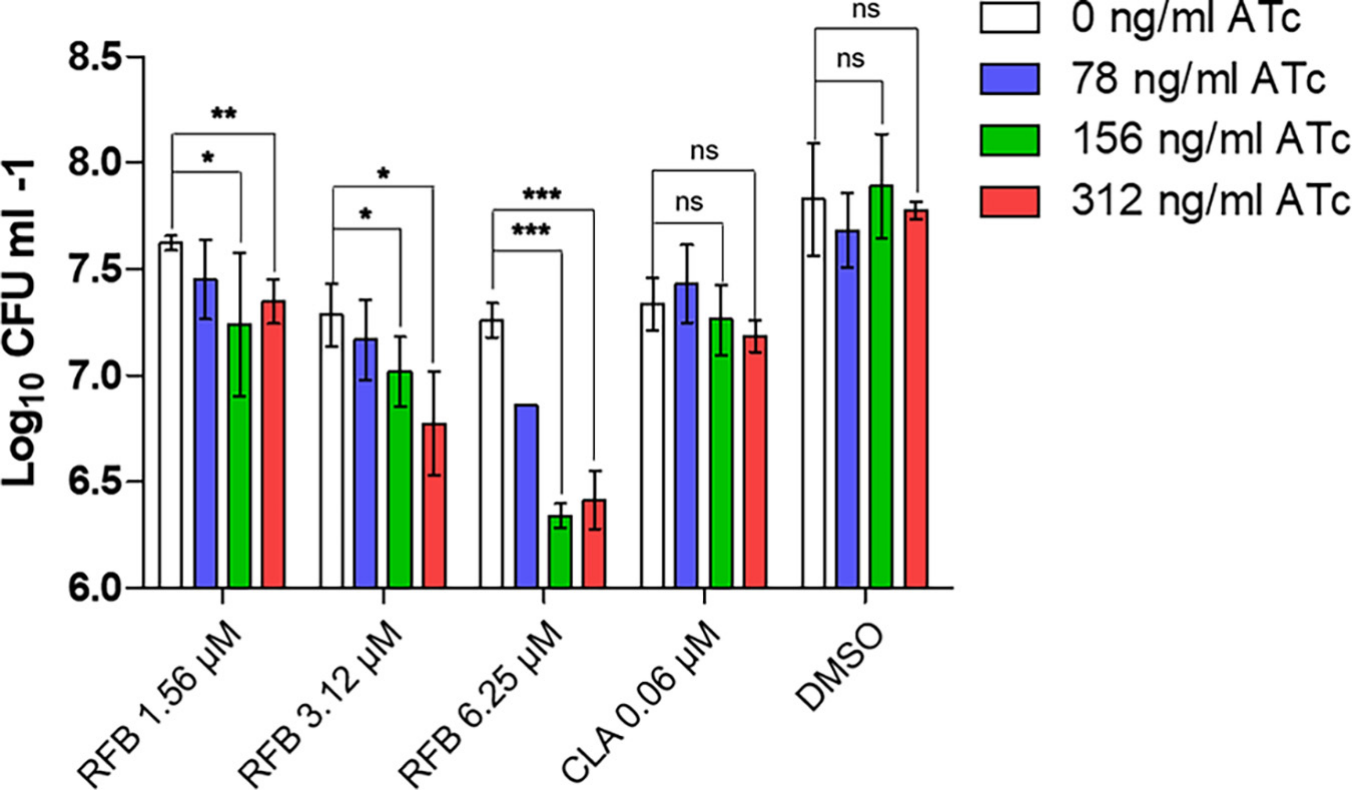
*MAB_0055c*-transcriptional knockdown of *MAB_0055c*-sgRNA strains specifically enhanced their susceptibility to RFB intracellularly. Changes in RFB intracellular antibiotic activity depending on ATc concentration were examined in bone-marrow-derived macrophages (BMDMs). BMDMs were infected with the *MAB_0055c*-sgRNA strain under different concentrations of ATc and then the intracellular activity of RFB was determined by CFU assay. CLA and DMSO were used as the negative control. Results are the mean ± SD of three replicates. Notably, an increase of ATc showed a decrease in intracellular bacterial growth at 1.56, 3.12, and 6.25 μM doses of RFB, and yet, CFU was not significantly changed in the CLA-treated or DMSO control groups. Statistical analysis was conducted using an unpaired *t* test, and the results are presented as means ± SD from the experiment, which was performed in triplicate. The significance levels are denoted; *, *P < *0.05; **, *P < *0.01; ***, *P < *0.001; ns, not significant.

Three different ATc concentrations (78, 156, and 312 ng/mL) that reduced the number of infected BMDMs by 22%, 26%, and 36% were selected for the next experiment. Using these concentrations, the ATc concentration-dependent changes in antibiotic activity within BMDMs were evaluated using a quantification of bacterial burden through CFU determination. The RFB and CLA concentrations required to inhibit ≥50% of bacterial growth in macrophages were used for this experiment (see Fig. S4 in the supplemental material). Using 3 different fixed ATc concentrations and MIC_50_ values of RFB and CLA, the MAB_0055c-specific RFB resistance in macrophages was evaluated. As shown in Fig. 5, RFB showed significant ATc concentration-dependent changes in antibiotic activity against the replication of the *MAB_0055c*-sgRNA strain in macrophages. Specifically, host cells treated with 312 ng/mL of ATc showed a more significant reduction in bacterial CFU than those treated with 78 and 156 ng/mL of ATc under fixed RFB concentrations (3.12 and 6.25 μM, respectively). For example, cells treated with 156 ng/mL of ATc suppressed approximately 1.5 log_10_ intracellular bacterial growth compared with that of cells treated with 0 ng/mL ATc under 6.25 μM RFB. However, this phenomenon was not observed in the CLA-treated groups or the dimethyl sulfoxide (DMSO) control, as both showed no ATc-dependent intracellular activity changes. Hence, this result indicates that inhibition of MAB_0055c by sgRNA specifically enhances the antibiotic activity of RFB against the *MAB_0055c*-sgRNA strain, not only *in vitro* but also intracellularly.

**FIG 6:**
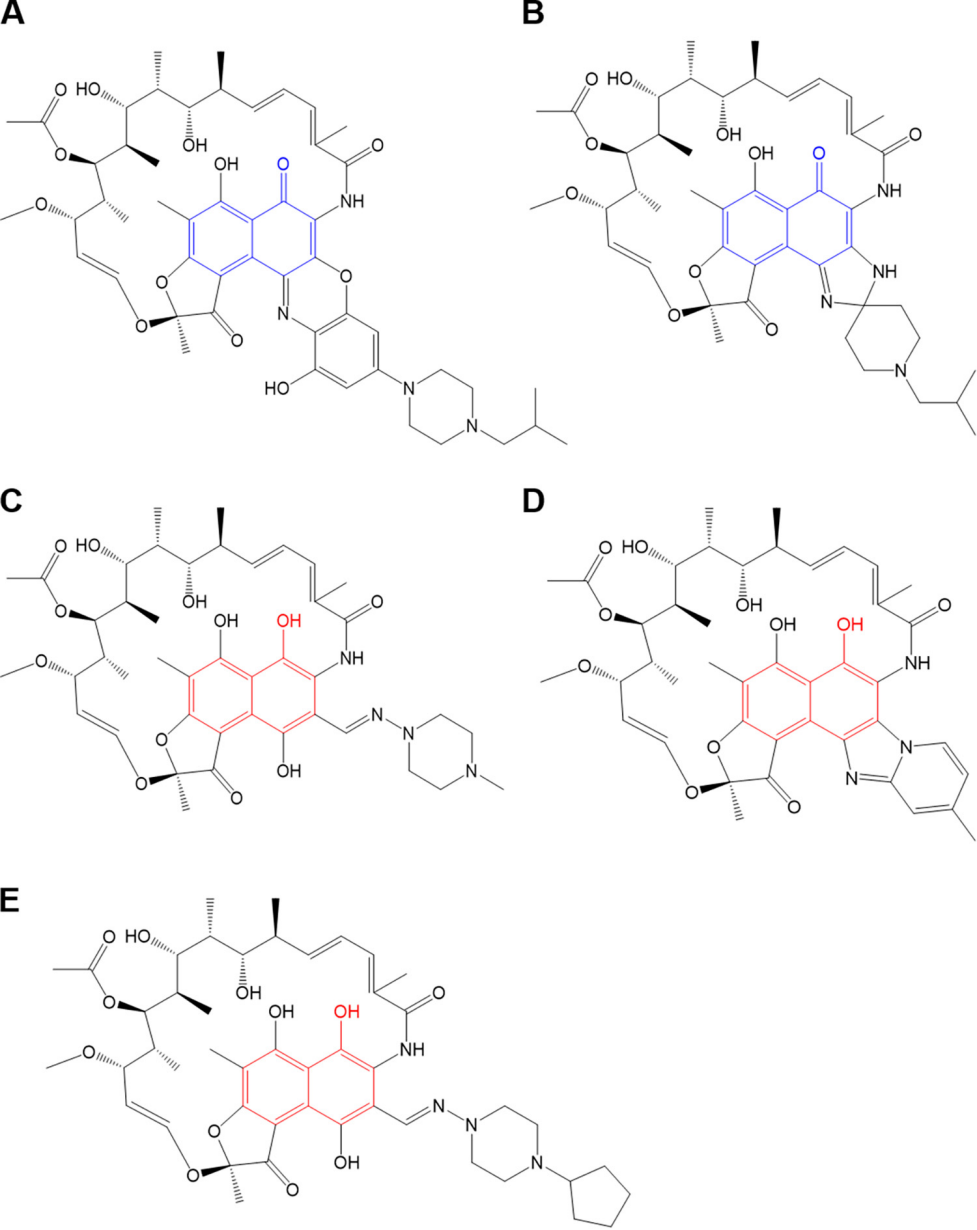
Chemical structures of rifamycin analogs. Rifalazil (A), RFB (B), RIF (C), rifapentine (D), and rifaximin (E). Blue and red indicate naphthoquinone and naphthohydroquinone cores, respectively.

**FIG 7:**
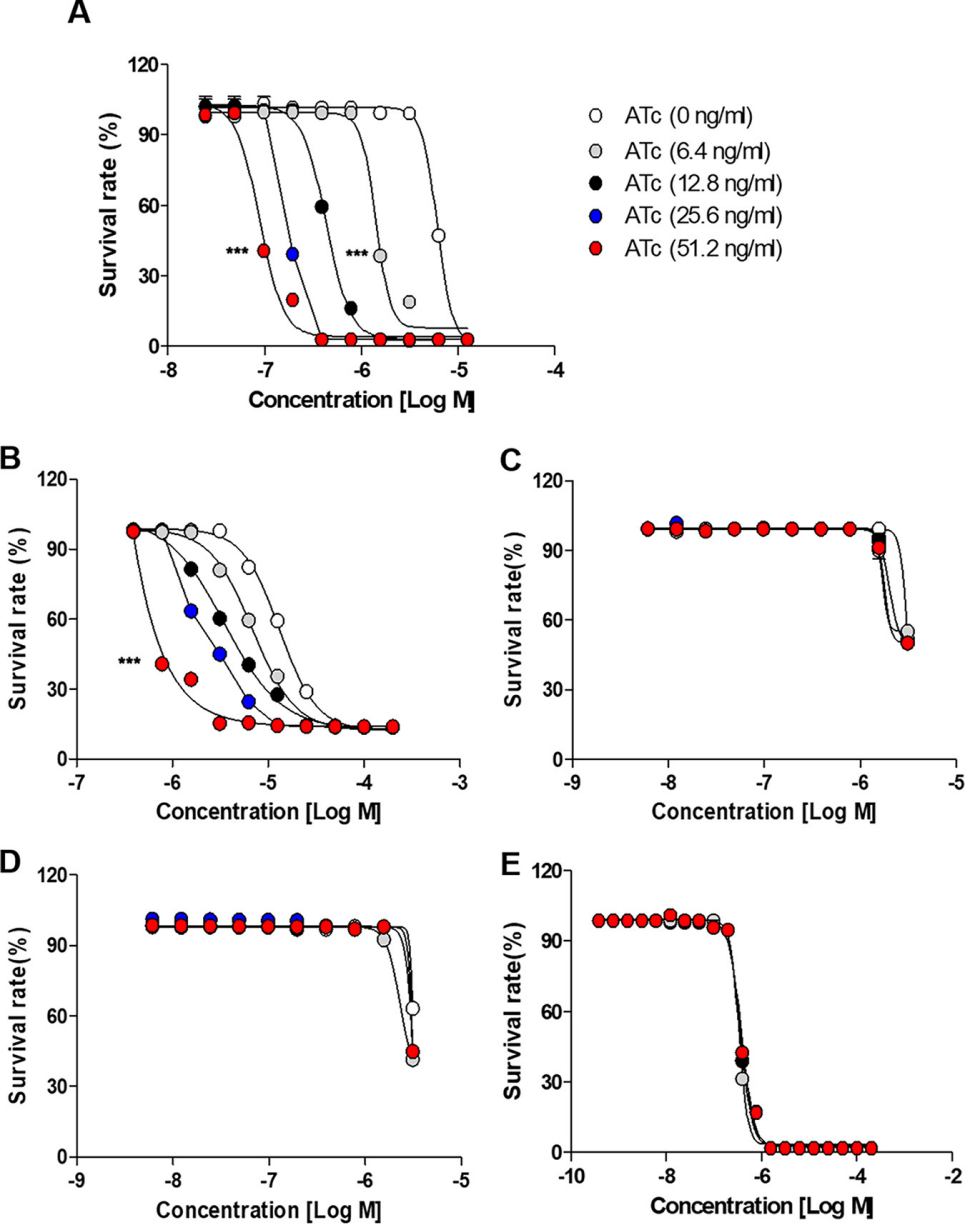
Interactions between MAB_0055c and structurally different rifamycin analogs under various concentrations of ATc. Several rifamycin analogs, either with or without a hydroquinone moiety, were administered to the *MAB_0055c*-sgRNA strain under various concentrations of ATc, and changes to their antibiotic activity were observed. RFB (A), rifalazil (B), RIF (C), rifapentine (D), and rifaximin (E). The survival of bacteria at each concentration was determined by calculating the ratio of relative light units (RLU) in the presence or absence of the inhibitor. The presented data represent the means of three independent experiments and are presented with standard deviation (SD) error bars. To determine statistical significance against the 0 ng/mL ATc control, a two-way ANOVA was performed. The results revealed a highly significant difference; ***, *P < *0.001.

RFB is a member of the rifamycin group, which includes rifamycin derivatives, such as RIF, RFB, rifapentine, rifalazil, and rifaximin (Fig. 6) (26). Among these rifamycin analogs, RIF, rifapentine, and rifaximin contain structural hydroquinone moieties at the C_1_ and C_4_ positions, while RFB and rifalazil do not. These hydroquinones are easily prone to autoxidation inside or outside the cell in the presence of oxygen and divalent metal cations, which results in a loss of antimycobacterial activity (10, 11). Currently, only RFB has been found effective against M. abscessus in an *in vivo* animal model (8, 27). Its lack of hydroquinone confers resistance to autoxidation, and consequently, it retains antibiotic activity against M. abscessus under oxidizing conditions (10, 11). Thus, the influence of MAB_0055c on rifamycin analogs that contain different moieties at the C_1_ and C_4_ positions was also evaluated. As demonstrated in Fig. 7, only rifamycin analogs that do not contain hydroquinones at the C_1_ and C_4_ positions showed changes in MIC_50_ in response to MAB_0055c inhibition. Specifically, increasing the dose of ATc from 6.4 to 51.2 ng/mL decreased the MIC_50_ of RFB against the *MAB_0055c*-sgRNA strain in dose-dependent manner. For example, supplementation of ATc at 51.2 ng/mL changed MIC_50_ values of RFB from 4.8 μM to 88.4 nM (Fig. 7A). Similarly, increasing the dose of ATc from 6.4 to 51.2 ng/mL gradually decreased the MIC_50_ of rifalazil against M. abscessus (Fig. 7B). However, other rifamycin analogs that have a hydroquinone at the C_1_ and C_4_ positions, such as RIF, rifapentine, and rifaximin, had no change in MIC_50_, even at the highest concentration of ATc (51.2 ng/mL) (Fig. 7C to E). Thus, it can be speculated that this structural uniqueness at the C_1_ and C_4_ positions is responsible for the interaction of the antibiotic with MAB_0055c.

Further characterization of specific gene functions, such as virulence, survival, and drug resistance, using genetic tools are crucial for combating M. abscessus infections. Here, CRISPRi-dCas9-based gene silencing technologies were used to knockout target genes in M. abscessus. This system employed a simple procedure to generate transcriptional knockdown strains that were rapidly obtained. Therefore, CRISPRi-dCas9 provides a simple genetic strategy for the validation of drug targets and mechanisms of action as well as for use in resistance studies necessary for drug discovery (20). Through the utilization of this technology, we have identified a functional association between MAB_0055c and rifamycin susceptibility. Additionally, we have demonstrated through CRISPRi-dCas9 conditional inhibition that MAB_0055c significantly contributes to the resistance of M. abscessus to RFB and rifalazil but not to other rifamycin analogs that contain a hydroquinone moiety. Moreover, our investigations have highlighted the crucial role of MAB_0055c in both *in vitro* and intracellular survival. This study presents the first finding of a connection between LTTR and antibiotic resistance in mycobacteria.

## MATERIALS AND METHODS

### Bacterial strains and culture conditions.

M. abscessus subsp. *abscessus* CIP 104536T S morphotype was a gift from Laurent Kremer (CNRS, IRIM, Universite’ de Montpellier, Montpellier, France). M. abscessus strains were grown in a Middlebrook 7H9 culture medium (Difco), supplemented with 10% albumin-dextrose-catalase (ADC; Difco), and 0.05% Tween 80 (Sigma). Middlebrook 7H10 solid culture medium containing 0.5% glycerol and 10% oleic acid-albumin-dextrose-catalase (OADC; Difco) was used for the CFU determination. To evaluate the MIC, the cation-adjusted Mueller-Hinton (CAMH) medium (Sigma, St. Louis, MO) supplemented with 20 mg/L calcium chloride (Sigma) and 10 mg/L magnesium chloride (Sigma) was used. All cultures were maintained at 37°C while shaking at 180 rpm. To generate mWasabi protein-expressing M. abscessus and the *MAB_0055c*-sgRNA strain, the plasmid pTEC15 (no. 30174; Addgene) was electroporated into M. abscessus and *MAB_0055c*-sgRNA, respectively, with hygromycin (500 μg/mL) for selection, as described previously (28, 29).

### Chemicals.

Clarithromycin (CLA), rifabutin (RFB), rifapentine (RFP), rifalazil (RFL), rifampicin (RIF), and moxifloxacin were purchased from Sigma-Aldrich (St. Louis, MO). Tigecycline (TGC) and bedaquiline (BDQ) were bought from Adooq Bioscience (Irvine, CA).

### Construction of *MAB_0055c*-sgRNA strain.

To achieve the *MAB_0055c*-sgRNA strain, the plasmid pLJR962 (no. 115162; Addgene) was used as the vector for CRISPRi in M. abscessus. Geneious 9.1.5 software was used to construct sgRNAs against *MAB_0055c*. The forward and reverse 26-nucleotide sgRNAs were ordered from Macrogen (Seoul, South Korea). The pLJR962 was linearized by BsmBI digestion (Enzynomics, Daejeon, South Korea) and gel purified. The forward and reverse sgRNAs were then annealed and ligated into the digested pLJR962 by using T4 ligase (New England BioLabs). The pLJR962-sgRNA constructs were transformed into Escherichia coli DH5α, and the plasmids prepared by QIAprep spin miniprep kit (Qiagen) were electroporated into M. abscessus. Transformants were selected on plates containing kanamycin (500 μg/mL).

### MAB_0055c suppression strain validation by real-time quantitative PCR (qPCR).

Overnight cultures of pLJR962 M. abscessus transformants with or without target sgRNA strains (OD_600_, ~0.8) were inoculated into Middlebrook 7H9 liquid medium (Difco), supplemented with 10% albumin-dextrose-catalase (ADC; Difco) and 0.05% Tween 80 (Sigma) with and without 150 ng/mL anhydrotetracycline (ATc) and incubated at 37°C for 3 days. Bacteria were centrifuged for 10 min at 12,000 rpm at 4°C, and supernatants were removed completely. The bacterial pellet was resuspended in 1 mL of TRIzol (Invitrogen), transferred to 2-mL disruption tubes (Lysing Matrix B; MP Biomedicals) for lysis using a FastPrep-24 5G (MP) instrument with 3 cycles of 6.5 m · s^−1^ for 30 s, and chilled on ice after each cycle. A total of 300 μL chloroform was added to each sample, mixed, and centrifuged for 15 min at 12,000 rpm and 4°C. RNA was recovered from the aqueous layer and purified after DNase digestion in-column using the RNase-free DNase set (Qiagen, Venlo, Netherlands) according to the manufacturer’s instructions. A NanoDrop 2000c (Thermo Fisher Scientific) instrument was used to determine RNA concentrations. The primer sequences for reverse transcription-quantitative PCR (qRT-PCR) are listed in Table 1. cDNA was prepared for qRT-PCR using the SensiFAST SYBR no-ROX one-step kit (Bioline). For the one-step reaction, the master mix was prepared using 2× SensiFAST SYBR no-ROX one-step mix, reverse transcriptase, RiboSafe RNase inhibitor, and primers (10 μM final concentration). The master mix and RNA sample were then added to each tube (20-μL total volume) and contents were mixed. The reaction was carried out on a QuantStudio 3 real-time PCR system (Thermo Fisher Scientific). The qRT-PCR step involved incubation at 45°C for 10 min. The PCR cycling conditions included an initial denaturation of 95°C for 2 min followed by 40 cycles of 95°C for 5 sec and 60°C for 20 sec. Relative quantification was performed using the 2^−ΔΔ^*^CT^* method, and the relative expression was calculated as the ratio between the mean threshold cycle (*C_T_*) values of the target genes and the reference gene (16S rRNA) in each stimulated sample relative to a reference sample.

**TABLE 1:** Primers used in this study

Oligonucleotide name	Sequence (5′–3′)	Purpose
*MAB_0055c*_sgRNA_F	GGGAGGGCATCCAGATCGCGCAGGGCCAGGC	sgRNA
*MAB_0055c*_sgRNA_R	AAACGCCTGGCCCTGCGCGATCTGGATGCCC	sgRNA
*MAB_0055c*_qRTPCR_F	TATTCACCGAGACTCTGCGC	Real-time qPCR
*MAB_0055c*_qRTPCR_R	AATAGGCGTCGATGTCCTGC	Real-time qPCR
*MAB_0055c*_qRTPCR_F	TCCGACGATCTACCGCTTCT	Real-time qPCR
*MAB_0055c*_qRTPCR_R	CACCTTCCGTACGTCCAGTT	Real-time qPCR
*MAB_0055c*_qRTPCR_F	CGGTTCCGGTCATCACGTAT	Real-time qPCR
*MAB_0055c*_qRTPCR_R	ATTCCAGGCAGATCTTGGGC	Real-time qPCR

### Resazurin microtiter assay (REMA).

Overnight cultures of pLJR962 M. abscessus transformants with or without target sgRNA strains (OD_600_, ~0.8) were collected and adjusted to an OD_600_ of 0.05 in wells of a 96-well microtiter plate. Two-fold serial dilutions of antibiotic or ATc were prepared. Plates were then incubated at 37°C for 3 days prior to supplementing 40 μL of the resazurin (Sigma, St. Louis, MO) per well. The SpectraMax M3 multi-mode microplate reader (Molecular Devices, Sunnyvale, CA) was used to measure fluorescence. MIC_50_ values were determined by fitting the curves with a sigmoidal dose-response using the GraphPad Prism software (version 6.05; San Diego, CA).

### Bone-marrow-derived macrophage infection and intracellular drug activity assessment.

Bone-marrow-derived macrophages (BMDMs) were isolated by flushing the femur and tibia from 6-week-old C57BL/6 mice (KOATECH). For differentiation, cells were cultured in Dulbecco’s modified Eagle’s medium (DMEM; Welgene) with 10% fetal bovine serum (FBS; Welgene), GlutaMax (35050-061; Gibco), and penicillin-streptomycin (15140-122; Gibco) in the presence of recombinant murine macrophage colony-stimulating factor (M-CSF; JW-M003-0025; JW CreaGene) at 37°C for 5 to 6 days. All mouse experiments conducted in this study were approved by the institutional animal care and use committee of Gyeongsang National University (GNU-220620-M0071).

To infect mWasabi protein-expressing M. abscessus transformants that either contained or lacked target sgRNA strains, each transformant was grown with and without ATc until the late log phase (OD_600_, ~0.8). BMDMs also were seeded in 96-well plates at 7 × 10^5^ cells/well for infection. Cell culture medium in 96-well plates was replaced with M. abscessus-containing medium at a multiplicity of infection of 1. After a 3-h infection, extracellular bacteria in the medium were eliminated by using amikacin (250 μg/mL) for 2 h. The amikacin-containing medium was then removed, and the cells were washed three times with phosphate-buffered saline (PBS; Gibco). Subsequently, the cells were treated with various concentrations of compounds for 3 days. For example, RFB and CLA were each subjected to 2-fold serial dilution, with RFB being diluted from a starting concentration of 25 μM to a final concentration of 0.19 μM and CLA being diluted from a starting concentration of 5 μM to a final concentration of 0.002 μM.

For visualization, cells were stained with SYTO 60 red fluorescent nucleic acid stain (Invitrogen). Fluorescent images were acquired by the ImageXpress Pico automated imaging system (Molecular Devices) and analyzed by CellReporterXpress image acquisition and analysis software (Molecular Devices) to quantify the total cell numbers and infected cell numbers.

A CFU assay was also conducted to titrate intracellular bacteria. The cells were lysed with 1% SDS (151-21-3; Generay Biotechnology) to release intracellular bacteria. The lysates were serially diluted with PBS. And then, each bacterial dilution was plated on 7H10 supplemented with appropriate antibiotics. After at least 3 days of incubation, bacterial colonies were counted.
