# Supplementary material for: CRISPR Interference-Based Inhibition of MAB_0055c Expression Alters Drug Sensitivity in Mycobacterium abscessus
Source: Microbiol Spectr. 2023 May 9;11(3):e00631-23. doi: 10.1128/spectrum.00631-23 (PMC10269454; doi:10.1128/spectrum.00631-23)
Supplement: Supplemental file 1 — Supplemental material. Download spectrum.00631-23-s0001.pdf, PDF file, 0.5 MB [file spectrum.00631-23-s0001.pdf]

Figure S1. *MAB_0055c*, *MAB_0054c*, and *MAB_0056c* gene expressions in an empty vector control and MAB0055c-sgRNA strain were observed after ATc treatment.

dCas9Sth1 can mediate the knockdown of *MAB_0055c* (+ATc ; 150 ng/ml) *in vitro* in a cultured medium (A). The expression of upstream (B) and downstream (C) genes remained unaltered. Gene knockdown was quantified by real-time qPCR; Error bars are confidence intervals of three technical replicates. The 16S rRNA gene was used as an internal standard. Statistical analysis was conducted using an unpaired t-test, and the results are presented as means ± SD from the experiment, which was performed in triplicate. Results are expressed as means ± standard deviations of the data from three experiments performed in triplicate. $^{**}P < 0.01$; ns is not significant.

Figure S2. Interactions between ATc and AMK, TGC, and CFX and their effects on growth of the *Mab*_0055c-sgRNA strain.

The effect of ATc (150 ng/ml) on the activity of AMK, TGC, and CFX against *MAB_0055c*-sgRNA strains was assessed by measuring bacterial growth on agar plates. Aliquots of each culture were subjected to 10-fold serial dilution and spotted onto 7H10 agar plates containing either (+) or (-) ATc. Bacterial growth was monitored to determine changes in the antibiotics' activity in the presence or absence of ATc.

Figure S3. Intracellular survival of *M. abscessus* and the Mab_0055c-sgRNA strain in the presence of various ATc concentrations.

Macrophages infected with mWasabi protein-expressing *M. abscessus* (A) and the Mab_0055c-sgRNA strain (B) were exposed to various ATc concentrations for 3 days, respectively. Blue squares indicate % of cells infected with bacteria and grey squares indicate number of cells stained by syto60. The CellReporterXpress® image acquisition and analysis software was used to quantify several different parameters, including the number of host macrophages, percentage of infected cells, and total fluorescence intensity.

Figure S4. Intracellular antibiotic activity of RFB and CLA.

M. abscessus empty vector control-infected mBMDMs were treated with various doses of RFB (A) and CLA (B) for 3 days. RFB and CLA effectively inhibited intracellular bacterial growth, while

remaining not toxic to cells at the drug concentrations tested. Macrophages were stained by syto60 for image analysis. The CellReporterXpress® image acquisition and analysis software was used to quantify several different parameters, including the number of host macrophages, percentage of infected cells, and total fluorescence intensity. Blue squares indicate % of cells infected with bacteria and grey squares indicate number of cells stained by syto60.
