# Supplementary material for: CRISPR Interference-Based Inhibition of MAB_0055c Expression Alters Drug Sensitivity in Mycobacterium abscessus
Source: Microbiol Spectr. 2023 May 9;11(3):e00631-23. doi: 10.1128/spectrum.00631-23 (PMC10269454; doi:10.1128/spectrum.00631-23)

# Supplementary figure 1

**A**

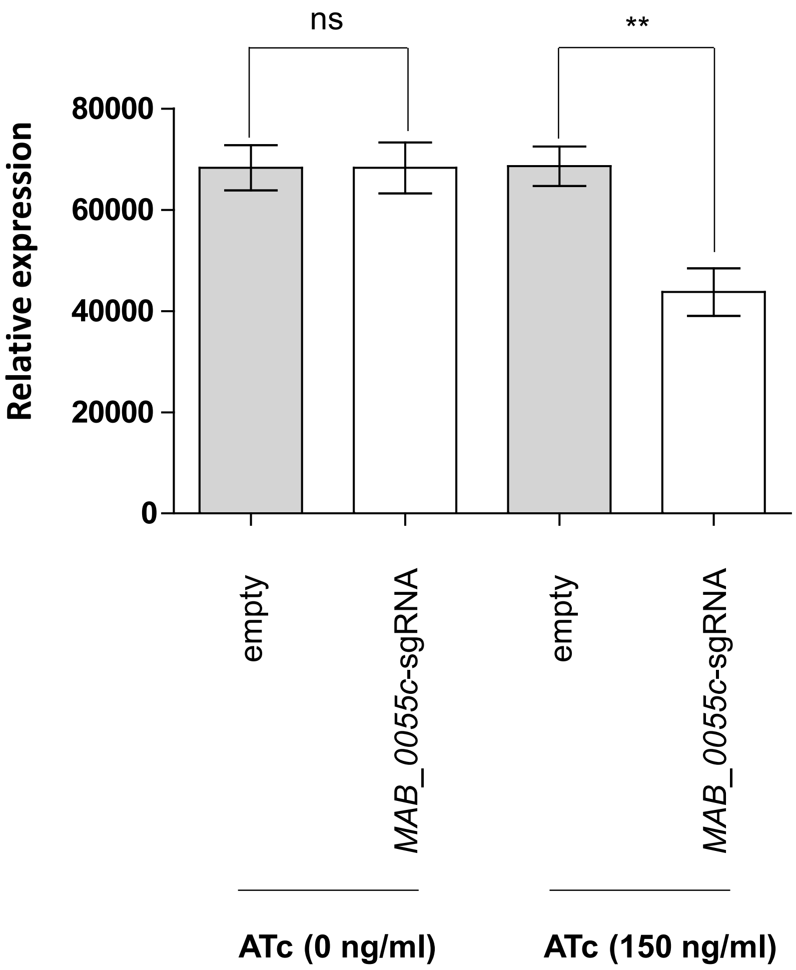


**B**

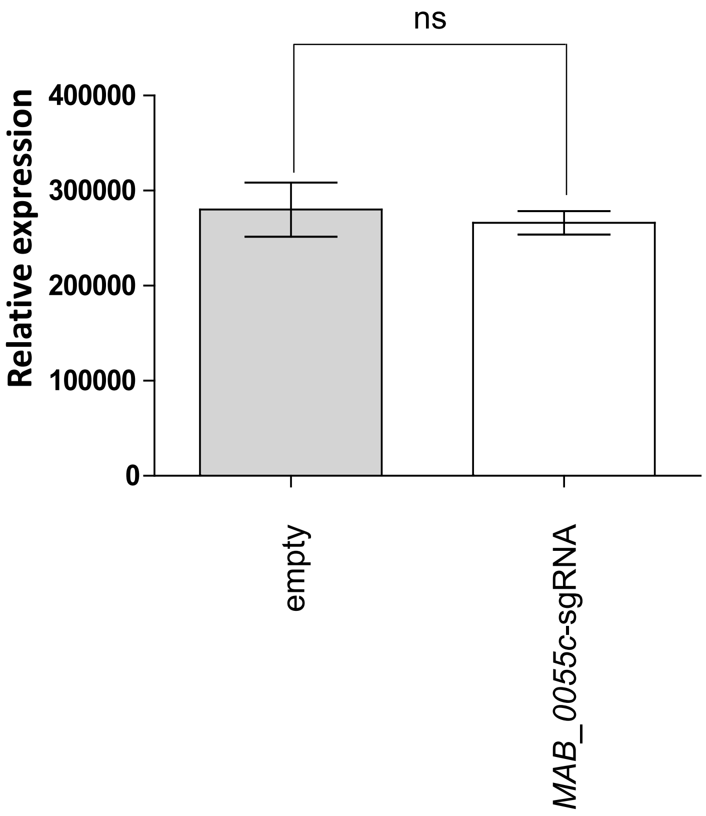


**C**

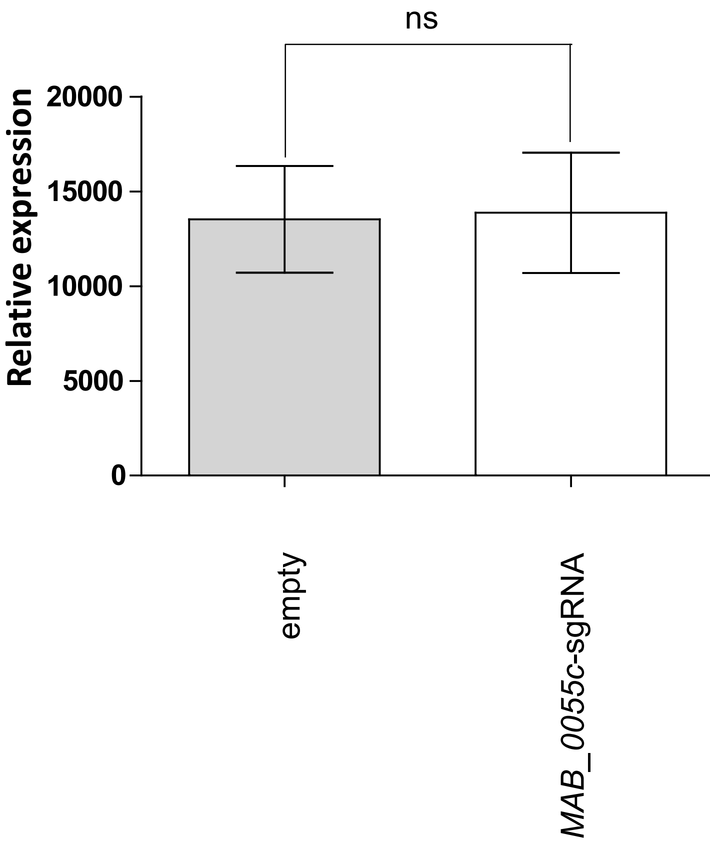

# Supplementary figure 2

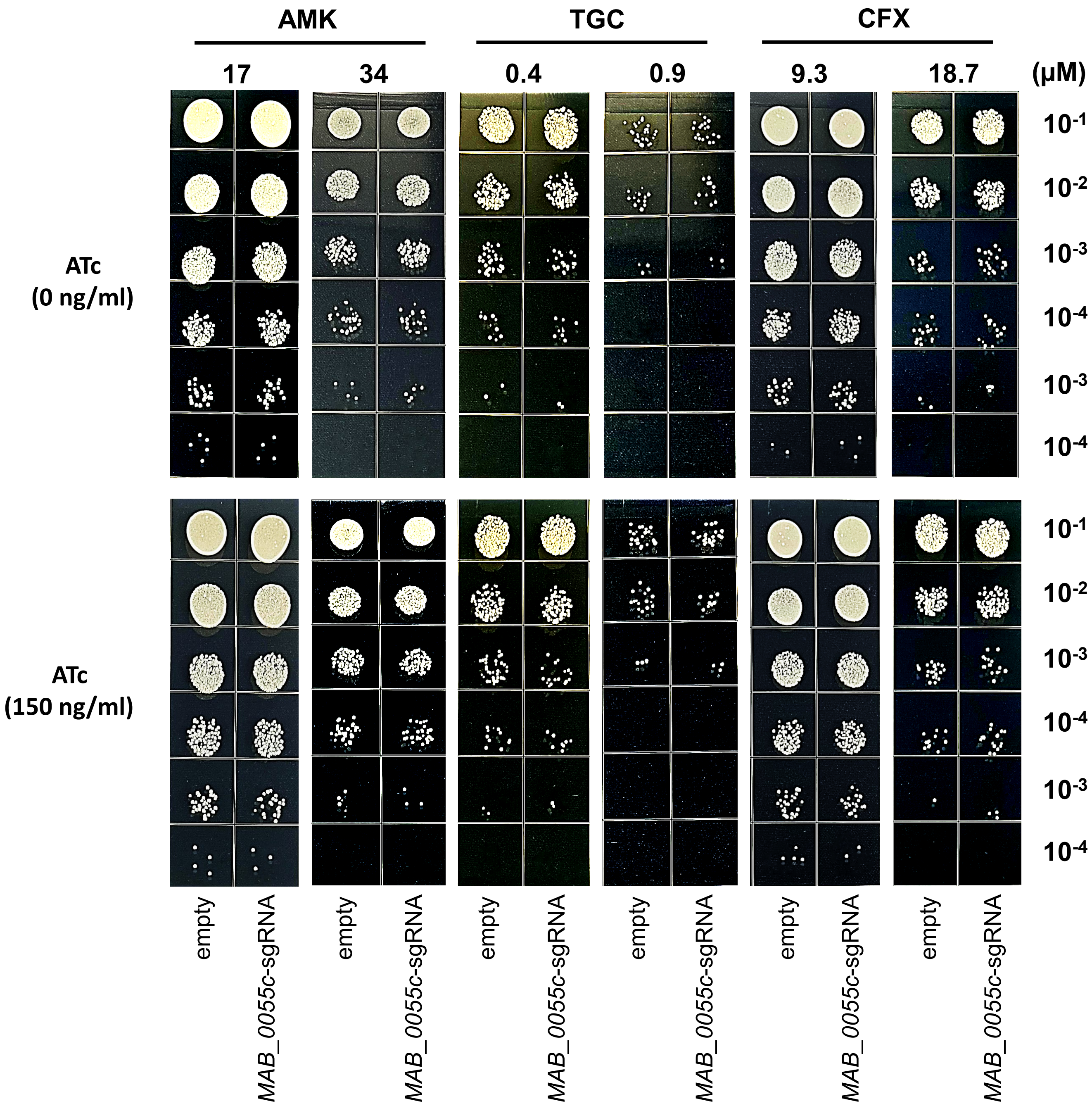

# Supplementary figure 3

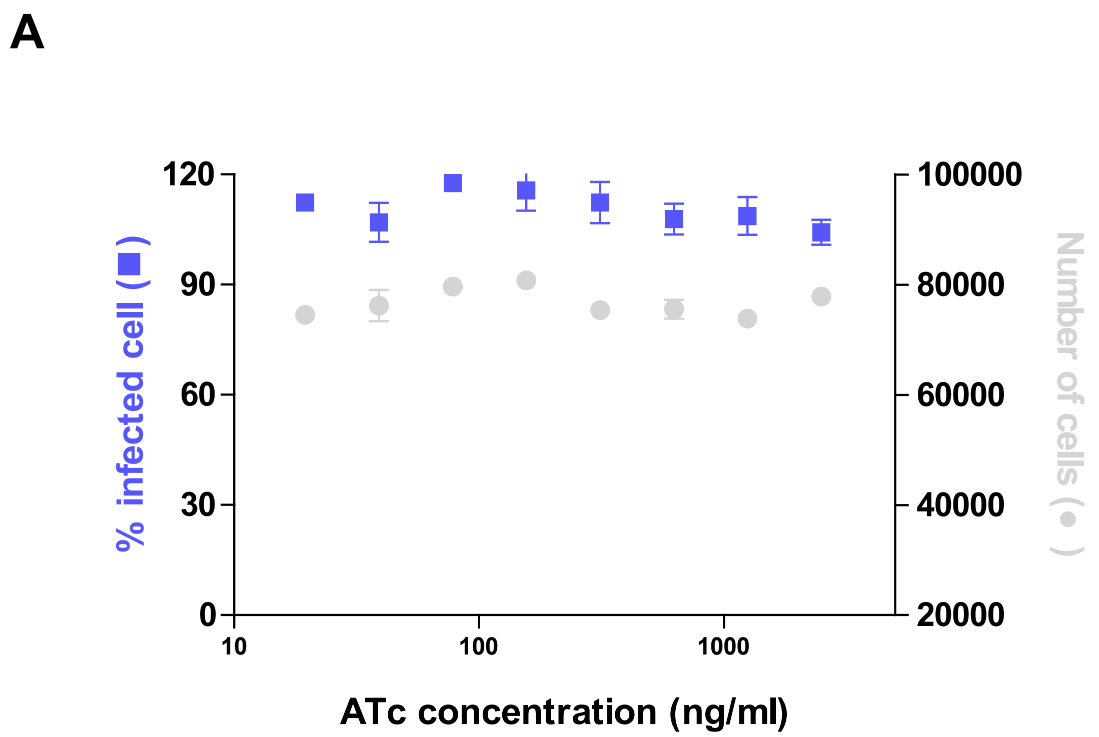


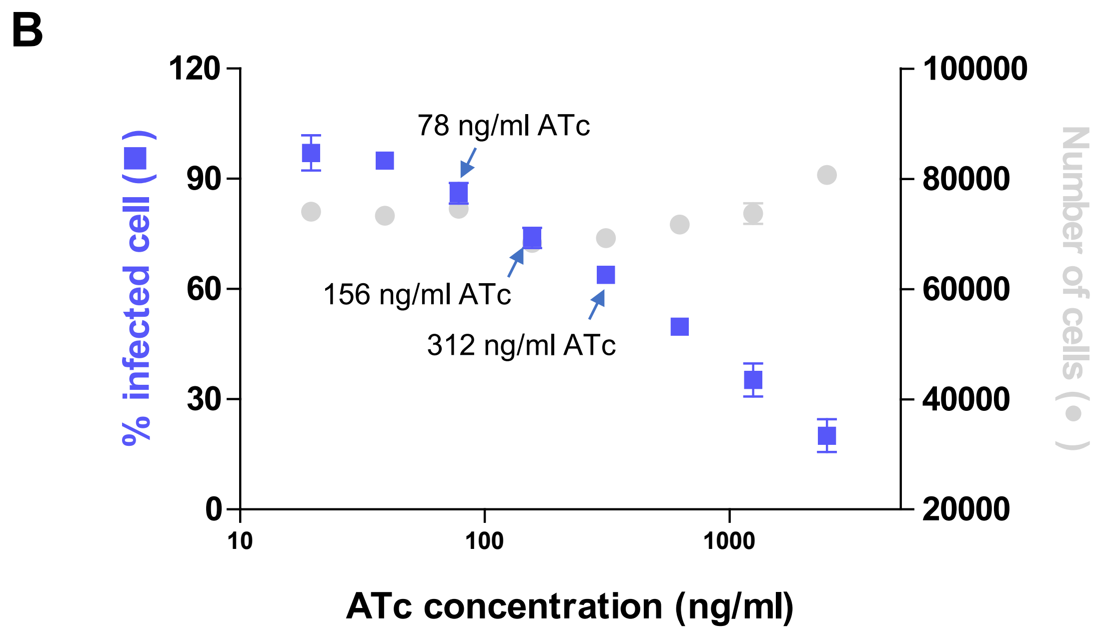

# Supplementary figure 4

A

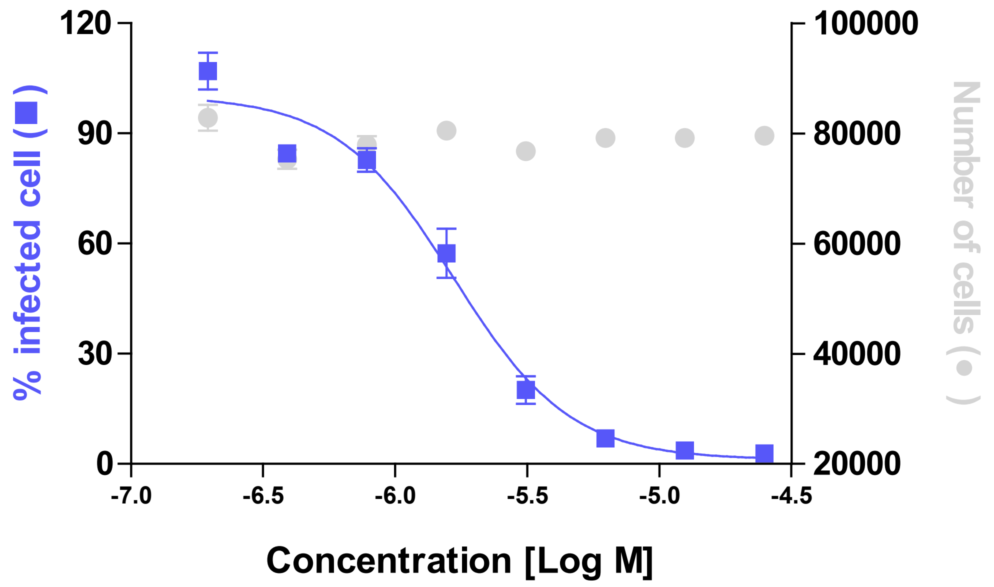


B

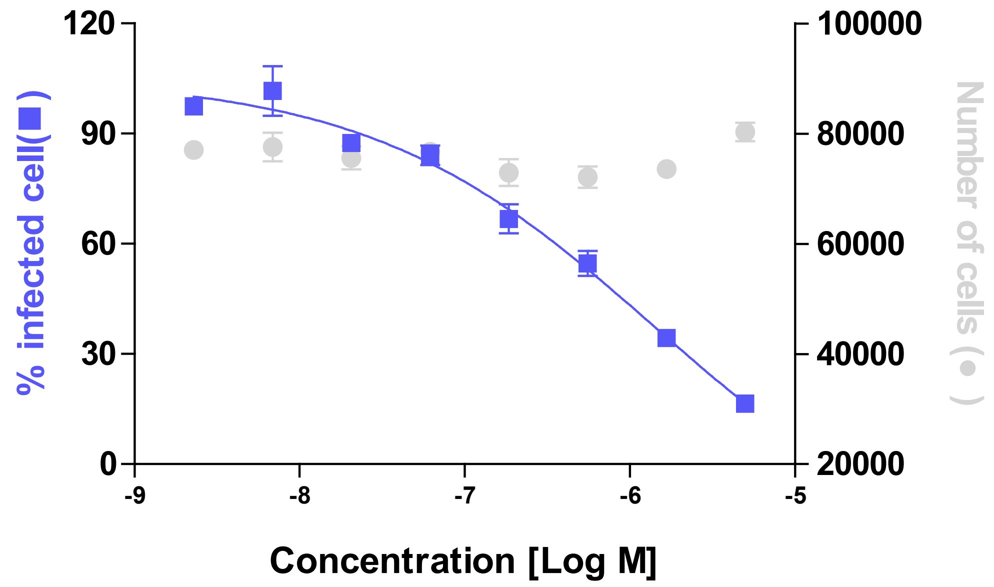

Supplement: Supplemental file 2 — Supplemental material. Download spectrum.00631-23-s0002.pdf, PDF file, 2.0 MB [file spectrum.00631-23-s0002.pdf]
